# Nb-doped NiO nanoflowers for nitrite electroreduction to ammonia

**DOI:** 10.1016/j.isci.2023.107944

**Published:** 2023-09-19

**Authors:** Ying Zhang, Yuying Wan, Xiaoxu Liu, Kai Chen, Ke Chu

**Affiliations:** 1 School of Materials Science and Engineering, Lanzhou Jiaotong University, Lanzhou 730070, China; 2 College of Science, Hebei North University, Zhangjiakou, Hebei 075000, China

**Keywords:** Electrochemistry, Applied chemistry, Energy materials

## Abstract

Electrocatalytic reduction of nitrite to ammonia (NO_2_RR) is considered as an appealing route to simultaneously achieve sustainable ammonia production and abate hazardous nitrite pollution. Herein, atomically Nb-doped NiO nanoflowers are designed as a high-performance NO_2_RR catalyst, which exhibits the highest NH_3_-Faradaic efficiency of 92.4% with an NH_3_ yield rate of 200.5 μmol h^−1^ cm^−2^ at −0.6 V RHE. Theoretical calculations unravel that Nb dopants can act as Lewis acid sites to render effective NO_2_^−^ activation, decreased protonation energy barriers, and restricted hydrogen evolution, ultimately leading to a high NO_2_RR selectivity and activity.

## Highlights

• Nb-doped NiO (Nb-NiO) is explored for electrochemical NO_2_^−^-to-NH_3_ reduction (NO_2_RR)
• Nb-NiO exhibits the FE_NH3_ of 92.4% with an NH_3_ yield rate of 200.5 μmol h^−1^ cm^−2^
• Nb-NiO can promote the NO_2_^−^ activation and decrease the protonation energy barriers

## Graphical abstract

**Figure:**
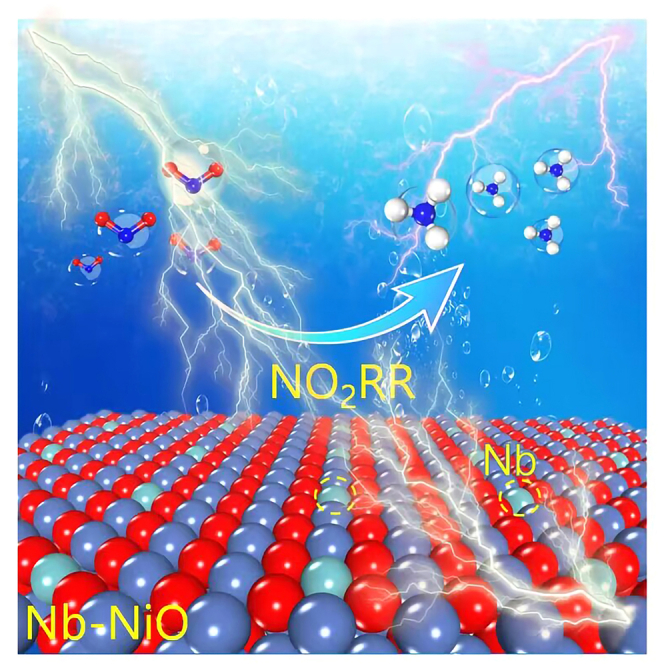

## Introduction

Ammonia (NH_3_) serves as a crucial chemical for fertilizer production and also as a storage medium for renewable energy.^1^^,^^2^^,^^3^ Electrochemical N_2_-to-NH_3_ reduction (NRR) in aqueous media is regarded as a prospective method for green NH_3_ production,^4^ but the NRR remains far from practical application, arising from the high dissociation energy of N≡N bond (927 kJ mol^−1^), the competitive hydrogen evolution reaction (HER), and the low N_2_ solubility.^5^^,^^6^^,^^7^^,^^8^^,^^9^^,^^10^ Nitrite (NO_2_^−^), a widespread N− pollutant, is extremely harmful to human health and ecological environment.^11^^,^^12^^,^^13^^,^^14^ Since NO_2_^−^ possesses a weaker N=O bond dissociation energy (204 kJ mol^−1^) and a higher water solubility, electrocatalytic NO_2_^−^-to-NH_3_ reduction (NO_2_RR) via a direct six-electron transfer process is considered as an attractive approach to simultaneously achieve effective ammonia production and abate hazardous nitrite pollution.^15^^,^^16^^,^^17^^,^^18^^,^^19^ However, developing NO_2_RR catalysts with high selectivity and activity remains a grand challenge.

As electron-deficient centers, Lewis acid sites possess empty orbitals capable of interacting with the electron lone pair of Lewis base NO_2_^−^ species,^20^ facilitating the activation and dissociation of NO_2_^−^. Besides, owing to the strong electrostatic repulsion effect, the adsorption of H atoms can be effectively impeded on Lewis acid sites,^21^ leading to an inhibited HER process. Therefore, the construction of Lewis acid sites on catalysts offers an efficient method for potentially achieving active and selective NO_2_RR. Extensive research has indicated that incorporating metal dopants into transition metal oxides is an effective method to create Lewis acid sites because their strong electronic interactions lead to charge redistribution on different metal atoms.^20^^,^^21^^,^^22^^,^^23^^,^^24^^,^^25^ Particularly, when metal dopants exist in isolated single-atom form, abundant Lewis acid sites would be generated,^26^^,^^27^^,^^28^ which are greatly favorable for the maximized Lewis acidity and largely expedited NO_2_RR activity.

Nb is known to possess a prominent Lewis acidity due to the existence of holes in its d orbitals.^29^ Nb-based catalysts have also been demonstrated to show high catalytic activity in N-cycle electrocatalytic reactions toward the ammonia electrosynthesis.^30^^,^^31^^,^^32^ Therefore, in this work, atomically Nb doped in NiO (Nb−NiO) nanoflowers are designed as a high-performance NO_2_RR catalyst, which exhibits a fascinating NO_2_RR performance with the highest FENH_3_ of 92.4% and NH_3_ yield rate of 200. 5 μmol h^−1 cm−2^ at −0.6 V, outperforming pristine NiO and many other reported NO_2_RR catalysts. Theoretical calculations reveal the pivotal role of Lewis acid Nb dopants in facilitating the activity and selectivity of NO_2_RR process.

## Results and discussion

**Figure 1:**
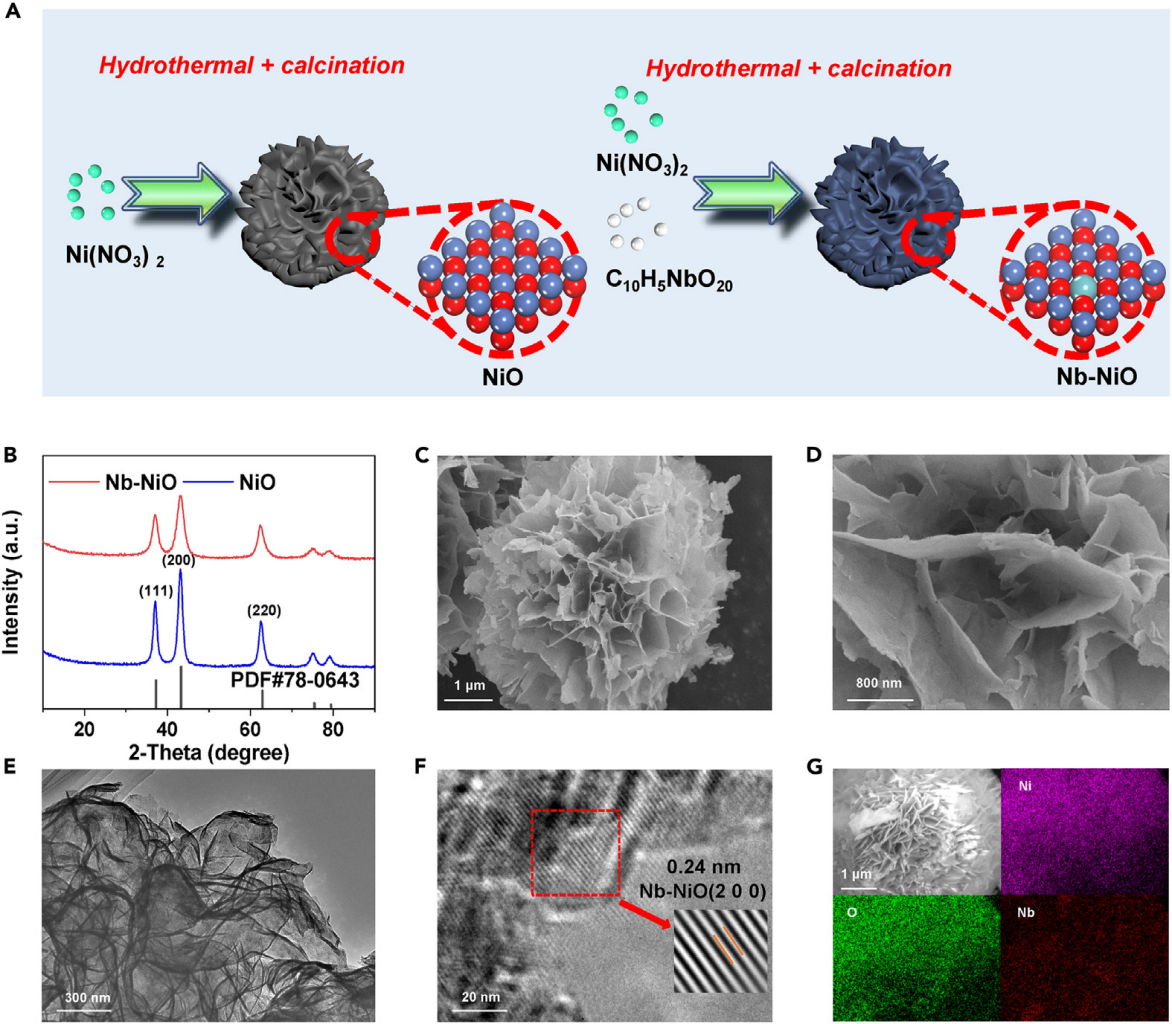
Morphology characteristics of Nb−NiO (A) Schematic diagram of the preparation route of NiO and Nb−NiO. (B−G) Characterizations of as-prepared Nb−NiO: (B) XRD patterns, (C and D) SEM images, (E) TEM image, (F) HRTEM image, (G) Elemental mapping images.

The synthesis of Nb−NiO nanoflowers is conducted by the combined hydrothermal and calcination methods (Figure 1A). The X-ray diffraction (XRD) patterns of both pristine NiO and Nb−NiO (Figure 1B) show the characteristic diffraction peaks of cubic NiO (No. 78−0643).^33^ A detailed inspection reveals that Nb−NiO delivers a slightly lower peak intensity and wider full width at half maximum compared to pristine NiO, arising from the incorporation of Nb dopants in Nb−NiO (Figure S1). Representative scanning electron microscopy (SEM) (Figures 1C and 1D) image of Nb−NiO shows a typical nanoflower structure comprising many vertically aligned nanosheets, similar to that of original NiO (Figure S2A). The thin nanosheet feature of Nb−NiO (Figure 1E) and NiO (Figure S2B) can be further revealed by the transmission electron microscopy (TEM) image showing clear wrinkles and corrugations. In addition, the high-resolution transmission electron microscopy (HRTEM) image exhibits a clear lattice fringe of 0.24 nm, correlating well with (200) crystallographic plane of cubic NiO (Figure 1F). Elemental mapping images (Figure 1G) unveil that Nb dopants are uniformly dispersed over the whole surface of Nb−NiO nanoflowers.

**Figure 2:**
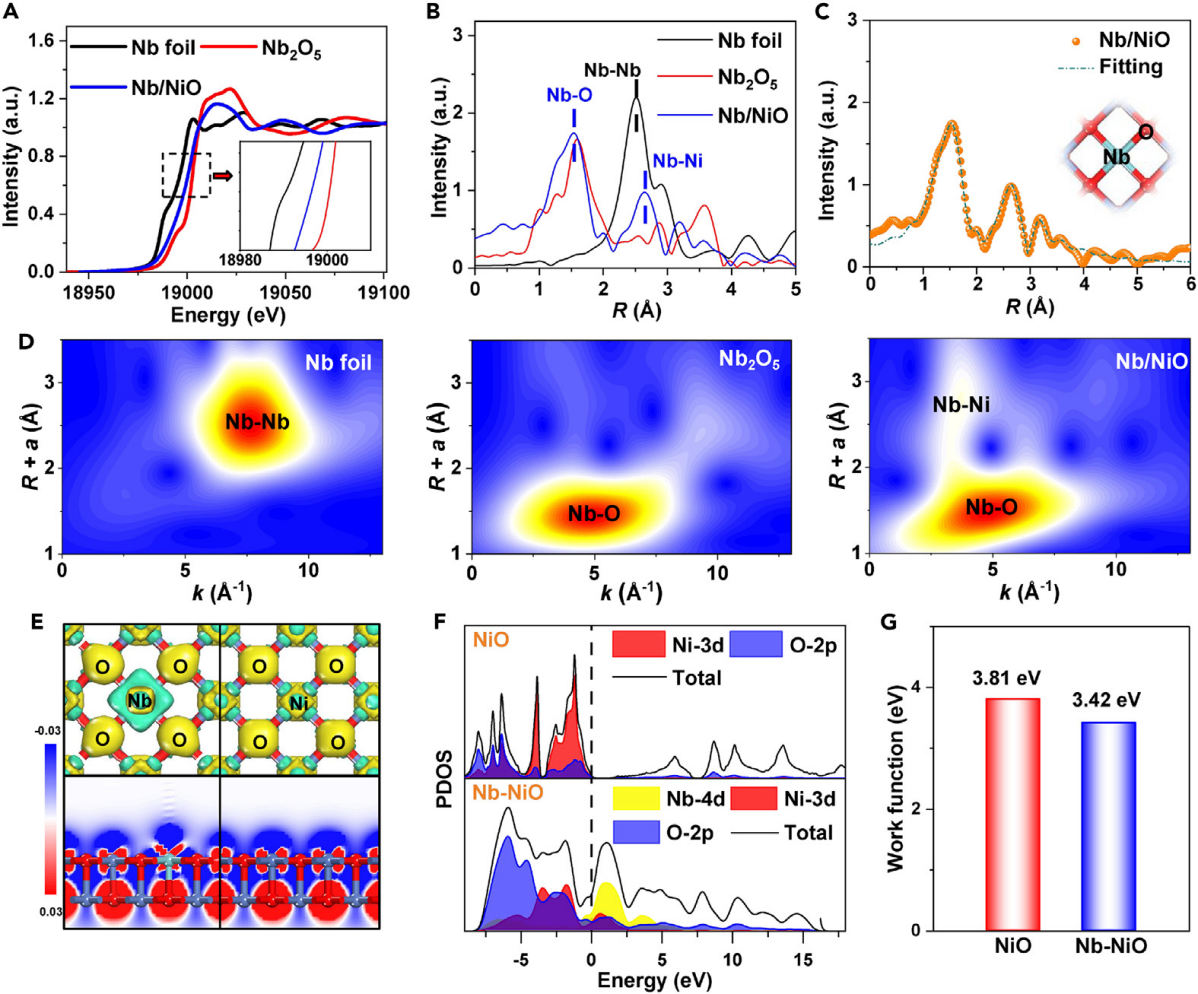
Structural characteristics of Nb−NiO (A, B, and D) (A) Nb K−edge XANES, (B) EXAFS spectra, and (D) WT profiles of Nb−NiO, Nb foil and Nb_2_O_5_. (C) EXAFS fitting analysis of Nb−NiO. (E) Charge density difference (top half) and electron location function (bottom half), yellow and red: charge accumulation, cyan and blue: charge depletion. (F and G) (F) PDOS profiles and (G) calculated work functions of NiO and Nb−NiO.

The X-ray absorption spectroscopy (XAS) characterizations are conducted to evaluate the coordination environment of Nb−NiO. The Nb K−edge X-ray absorption near edge structure (XANES) spectra (Figure 2A) show that the absorption edge of Nb-NiO is situated between Nb foil and Nb_2_O_5_, indicating that Nb dopants are in oxidation state. Linear XANES fitting result reveals that the average Nb valence state is +3.4 (Figure S3). The Nb K−edge extended X-ray absorption fine-structure (EXAFS) spectrum of Nb−NiO (Figure 2B) reveals a dominant peak at 1.54 Å, which is assigned to Nb−O first coordination shell. The absence of Ni−Ni coordination bond confirms the automatically dispersed Nb dopants in Nb−NiO.^34^^,^^35^^,^^36^ Besides, the 2.65 Å peak is assigned to Nb−Ni second coordination shell. Similarly, the wavelet-transformed (WT, Figure 2D) profiles display that Nb−NiO exhibits two Nb−O and Nb−Ni intensity maxima. EXAFS fitting analysis shows that the isolated Nb atom coordinates with five adjacent O atoms to form geometric Nb_1_−O_5_ moiety (Figure 2C; Table S1).

Density functional theory (DFT) computations are performed to investigate the electronic structures of Nb−NiO. On the basis of XRD and HRTEM results, we select (200) plane of NiO slab for Nb−NiO structural modeling. As seen in Figure S4, by substituting a surface-exposed Ni atom with an Nb dopant, the resulting Nb−NiO shows a rather negative formation energy of −2.46 eV, suggesting that Nb dopant incorporated in NiO lattice is thermodynamically feasible.^37^ Charge density difference and electron location function (ELF, Figure 2E) maps of Nb−NiO exhibit the noticeable electron-deficient regions around Nb dopant. This can be further verified by the detailed charge analysis (Figure S5), in which Nb dopant (+1.12 |e|) is more positively charged than Ni (+0.77 |e|) and thus Nb dopants can serve as Lewis acid sites to activate and polarize NO_2_^−^ during the NO_2_RR process.^38^ The projected density of states (PDOS, Figure 2F) analysis displays that NiO possesses a distinct band gap, indicating its semiconducting nature. In stark contrast, introducing Nb dopant in NiO generates significant electronic states crossing the Fermi level, suggesting the metallic character and improved conductivity of Nb−NiO.^39^ Meanwhile, as shown in Figure 2G (Figure S6), the calculated work function (Φ) value of Nb−NiO is 3.42 eV, which is lower than that of NiO (3.81 eV). Thus, the proton-coupled electron transfer process and the electrocatalytic NO_2_RR kinetics can be significantly facilitated on Nb−NiO.^40^ Moreover, AIMD simulations of Nb−NiO display the equilibrium states of energy and temperature (Figure S7), signifying the high thermodynamic stability of Nb−NiO.^40^

**Figure 3:**
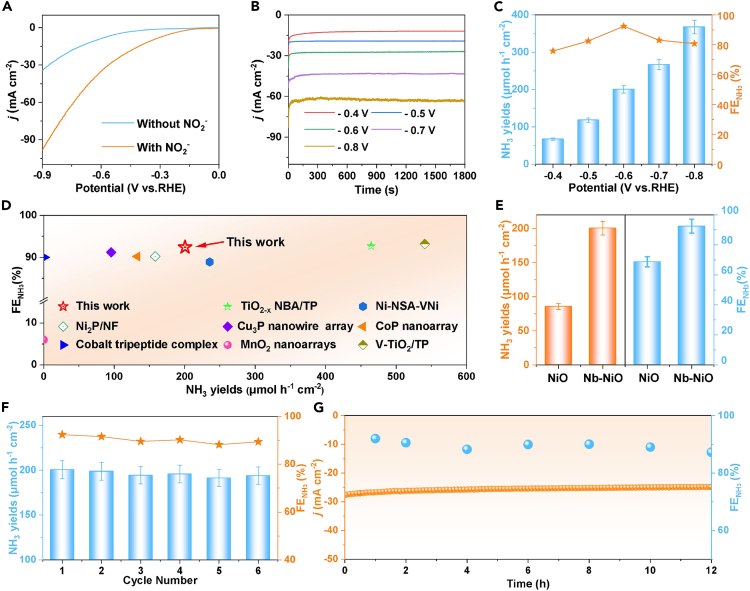
Electrochemical NO_2_RR tsts (A) LSV curves of Nb−NiO in various electrolytes. (B and C) (B) Chronoamperometry test of Nb−NiO at different potentials after 0.5 h electrolysis and (C) obtained NH_3_ yield rates and FE_NH3_. (D) Comparison of NH_3_ yield rates and FENH3 between Nb−NiO and reported NO2RR catalysts. (E) Comparison of the NO_2_RR performance between NiO and Nb−NiO at −0.6 V. (F and G) (F) Cycling and (G) long-term stability tests of Nb−NiO at −0.6 V.

Electrochemical NO_2_RR performance of Nb−NiO is evaluated in 0.5 M Na_2_SO_4_ + 0.1 M NaNO_2_ solution using an H cell based on a standard procedure flow chart (Figure S8).^14^ The produced liquid and gas products after NO_2_RR electrolysis are determined by colorimetric and gas chromatography methods (Figures S9−S11),^41^^,^^42^^,^^43^^,^^44^ respectively. The linear sweep voltammetry (LSV) curves of Nb−NiO are measured firstly (Figure 3A), and a significant increase in current density (*j*) is observed for NO_2_^−^-containing electrolyte compared to NO_2_^−^-free electrolyte, signifying the high NO_2_RR activity of Nb−NiO. Subsequently, the NO_2_RR performance of Nb−NiO is quantitatively evaluated at various potentials using the combined chronoamperometry (Figure 3B) and colorimetric tests. Figure 3C shows that Nb−NiO shows a maximum FENH_3_ of 92.4% at −0.6 V, with the corresponding NH_3_ yield rate reaching 200.5 μmol h^−1^ cm^−2^. Such NO_2_RR performance is better than that of most reported NO_2_RR catalysts as depicted in Figure 3D and Table S2. The controlled colorimetric measurements (Figure S12) and alternating experiments (Figure S13) attest that the generated NH_3_ is derived from the NO_2_RR electrolysis on Nb−NiO.^45^^,^^46^^,^^47^^,^^48^

Regarding the NO_2_RR selectivity, Nb−NiO exhibits fairly low Faradaic efficiencies (FEs) for H_2_, NH_2_OH, and N_2_H_4_ by-products relative to FENH_3_ (Figure S14), confirming a high NO_2_RR selectivity of Nb−NiO toward the NH_3_ generation. This finding can be further confirmed by the time-dependent NO_2_RR electrolysis (Figure S15), which shows a considerably decreased NO_2_^−^−N concentration coupled with a significantly increased NH_3_−N concentration as the electrolysis time increases. As a comparison, we evaluate the NO_2_RR performance of pristine NiO (Figure 3E), which exhibits much lower NO_2_RR activity and selectivity than Nb−NiO. Specifically, Nb−NiO outperforms pristine NiO by 2.3 and 1.3 times in NH_3_ yield rate and FENH_3_ at −0.6 V, respectively. Besides, Nb−NiO displays a higher electrochemical active surface area (ECSA, Figure S16) than NiO, while the catalyst performance normalized by ECSA (Figure S17) exhibits the same trend with Figure 3E, suggesting the high intrinsic activity of Nb−NiO toward the NO_2_RR. As for the electrocatalytic stability of Nb−NiO, slight changes in NH_3_ yield rates and FENH_3_ over six consecutive cycles can be seen, indicating an excellent cycling stability of Nb−NiO (Figure 3F). The long-term chronoamperometric experiment shows a negligible decline in current density and calculated FENH_3_ over 12 h continuous electrolysis (Figure 3G), substantiating the excellent long-term durability of Nb−NiO.^49^^,^^50^^,^^51^ After stability tests, Nb−NiO retains its original phase, morphology, and coordination structure (Figure S18), evidencing the high structural stability of Nb−NiO.^52^^,^^53^^,^^54^

**Figure 4:**
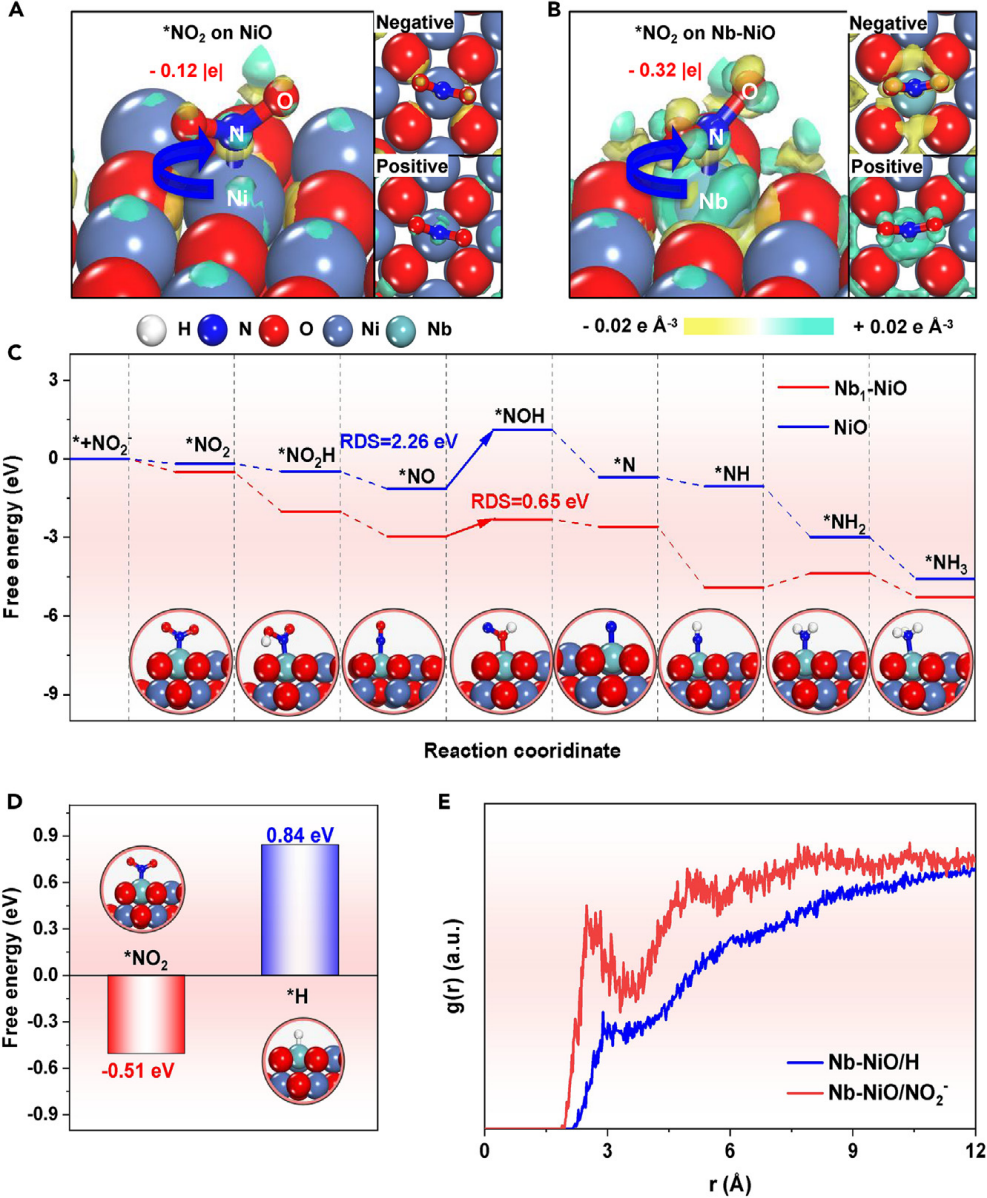
Theoretical analysis (A and B) Charge density difference plots of ∗NO_2_ on (A) NiO and (B) Nb−NiO. Yellow: charge accumulation, cyan: charge depletion. (C) Free energy profiles of NO_2_RR process on NiO and Nb−NiO. (D) Free energies of absorbed H and NO_2_^−^ on Nb-dopant site of Nb−NiO. (E) RDF curves of the interactions between Nb−dopant and NO_2_^−^/H^+^.

Theoretical calculations are conducted to elucidate the mechanism for the Nb-doping-induced enhanced NO_2_RR performance of Nb−NiO. Upon the NO_2_^−^ adsorption on Nb−NiO (Figure S19), the electron-deficient Nb dopant, as previously determined in Figure 2E, can serve as Lewis acid site to favorably absorb Lewis base NO_2_^−^, resulting in enhanced NO_2_^−^ activation and N=O bond dissociation.^55^^,^^56^^,^^57^^,^^58^ Charge density difference analysis (Figures 4A and 4B) reveals a remarkable Nb−∗NO_2_ electronic coupling where Nb dopant donates −0.32 |e| to ∗NO_2_, in stark contrast to −0.12 |e| for Ni−to−∗NO_2_ charge transfer. In addition, the free energy diagram (Figure 4C; Figure S20) presents that both Nb dopant of Nb−NiO and Ni site of NiO exhibit the same rate determining step (RDS) of ∗NO → ∗NHO.^59^ Nonetheless, Nb dopant exhibits a much reduced RDS energy barrier compared to Ni site (−2.26 eV). Besides, Nb dopant presents much lower free energies of all protonation intermediates than Ni site. Both findings demonstrate that Lewis acid Nb dopant serve as active site to significantly enhance the protonation energetics to boost the NO_2_^−^−to−NH_3_ conversion process on Nb−NiO.

Considering that HER is the main competing reaction of NO_2_RR,^60^ the HER activity of NO_2_RR-active Nb-dopant site is further investigated. As displayed in Figure 4D, the binding free energy of ∗H on Nb dopant of Nb−NiO is calculated as 0.84 eV, which is much positive than that of ∗NO_2_ (−0.51 eV), confirming an unfavorable HER performance of Nb−NiO, which is attributed to the Lewis acidity of Nb dopant capable of repelling the binding of positively charged H. Additionally, molecular dynamics (MD) simulations (Figure 4E) reveal that the snapshot after simulation (Figure S21) shows the aggregation of evident NO_2_^−^ on Nb−NiO, and the calculated radial distribution function (RDF) curves (Figure 4E) present a more intense Nb−NiO/∗NO_2_^−^ interaction compared to Nb−NiO/∗H interaction,^61^^,^^62^^,^^63^ further corroborating that Nb−NiO is able to selectively adsorb NO_2_^−^ and suppress H coverage, thus facilitating the boosted NO_2_RR and inhibited HER. These theoretical results reveal that the Lewis acid Nb dopant of Nb−NiO plays a crucial role in enhancing the efficient adsorption and activation of NO_2_^−^, boosting the protonation energetics and suppressing the HER, eventually leading to the high catalytic activity and selectivity of Nb−NiO for the NO_2_RR.

### Conclusion

Nb−NiO has been proved to be an efficient and robust NO_2_RR catalyst. Theoretical computations suggest that the enhanced NO_2_RR performance of Nb−NiO originates from the key role of Lewis acid Nb dopant in suppressing the HER and enhancing NO_2_^−^ activation and protonation. This work not only offers an in-depth understanding of the Lewis acid dopant-catalyzed NO_2_RR mechanism but also implies the great potential of constructing Lewis acid dopants in catalysts to achieve exceptional NO_2_^−^ electroreduction and beyond.

## STAR★Methods

### Key resources table

**Table:** 

REAGENT or RESOURCE	SOURCE	IDENTIFIER
**Chemicals, peptides, and recombinant proteins**		

NaNO_2_	Aladdin Co., Ltd.	7632-00-0
NaClO	Aladdin Co., Ltd.	7681-52-9
C_5_FeN_6_Na_2_O·2H_2_O	Aladdin Co., Ltd.	13755-38-9
H_2_O_2_	Beijing Chemical Corporation	7722-84-1
H_2_SO_4_	Beijing Chemical Corporation	7664-93-9
HCl	Beijing Chemical Corporation	7647-01-0
C_2_H_5_OH	Beijing Chemical Corporation	64-17-5
C_10_H_5_NbO_20_	Mclean Co., Ltd.	21348-59-4
Ni(NO_3_)_2_·6H_2_O	Sinopharm Chemical Reagent Co., Ltd.	13478-00-7

### Resource availability

#### Lead contact

Further information and requests for resources should be directed to and will be fulfilled by the lead contact, Dr. Ke Chu (chuk630@mail.lzjtu.cn).

#### Materials availability

This study did not generate new unique reagents. All chemicals were obtained from commercial resources and used as received.

### Method details

#### Synthesis of Nb−NiO

0.3 g Ni(NO_3_)_2_·6H_2_O and 0.32 g C_10_H_5_NbO_20_ were dispersed in 30 mL ethanol solution under stirring to form a transparent solution. Afterward, the solution was transferred into a 50 mL autoclave. After treatment at 150°C for 6 h, the light green precipitates were collected by centrifuging, washed with deionized water/ethanol and dried under vacuum overnight. The obtained precipitates were ground in an agate mortar and then transferred to a muffle furnace for calcination at 300°C for 4 h to obtain Nb−NiO. Pristine NiO was prepared by the same method as Nb−NiO by without addition of C_10_H_5_NbO_20_.

#### Electrochemical experiments

Electrochemical measurements were investigated with a CHI−760E electrochemical workstation using a conventional three−electrode cell. Nb−NiO coated on carbon cloth (1 × 1 cm^2^, 0.5 mg cm^−2^) was used as the working electrode, Ag/AgCl (saturated KCl) electrode was used as the reference electrode, and Pt foil was used as the counter electrode. All potentials were referenced to reversible hydrogen electrode (RHE) by following equation: *E* (V vs. RHE) = *E* (V vs. Ag/AgCl) + 0.198V + 0.059 × pH. The NO_2_RR measurements were carried out in 0.5 M Na_2_SO_4_ + 0.1 M NaNO_2_ electrolyte using an H−type two−compartment electrochemical cell separated by a Nafion 211 membrane. After each chronoamperometry test for 0.5 h, the produced NH_3_ and other possible by-product (N_2_H_4_) were analyzed by various colorimetric methods using UV-vis absorbance spectrophotometer (MAPADA P5), while the gas products (H_2_, NH_2_OH) were analyzed by gas chromatography (Shimadzu GC2010). The detailed determination procedures are given in our previous publication.^46^

Faradaic efficiency (FE) of NH_3_ generation was calculated by the following equation:

(Equation 1) FE=(6×F×c×V)/(17×Q)×100%

NH_3_ yield rate is calculated using the following equation:

(Equation 2) $${NH}_{3}\;yield\;rate=\left(c\times V\right)/\left(17\times t\times A\right)$$

where *c* (μg mL^−1^) is the measured NH_3_ concentration, *V* (mL) is the volume of the electrolyte in the cathode chamber (35 mL), *t* (s) is the electrolysis time, *A*(cm^−2^) is the surface area of CC (1 × 1 cm^2^), F (96500 C mol^−1^) is the Faraday constant, *Q* (C) is the total quantity of applied electricity.

#### Characterizations

X−ray diffraction (XRD) was conducted on a Rigaku D/max 2400 diffractometer. Scanning electron microscopy (SEM) was carried out on a ZEISS GeminiSEM−500 microscope. Transmission electron microscopy (TEM) and high-resolution transmission electron microscopy (HRTEM) were performed on a Tecnai G^2^ F20 microscope at an acceleration voltage of 200 kV.

## Data Availability

Data reported in this paper will be shared by the lead contact upon reasonable request. All original code is available in this paper’s supplemental information. Any additional information required to reanalyze the data reported in this paper is available from the lead contact upon reasonable request.
