# Supplementary material for: Nb-doped NiO nanoflowers for nitrite electroreduction to ammonia
Source: iScience. 2023 Sep 19;26(10):107944. doi: 10.1016/j.isci.2023.107944 (PMC10558769; doi:10.1016/j.isci.2023.107944)
Supplement: Document S1. Figures S1–S21 and Tables S1 and S2 [file mmc1.pdf]

iScience, Volume *26*

Supplemental information

Nb-doped NiO nanoflowers for nitrite electroreduction to ammonia

Ying Zhang, Yuying Wan, Xiaoxu Liu, Kai Chen, and Ke Chu

Calculation details

Cambridge sequential total energy package (CASTEP) module was applied for the density functional theory (DFT) calculations. The exchange−correlation function was utilized by Perdew−Burke−Ernzerhof (PBE) generalized gradient approximation (GGA) functional. The convergence criteria for structure optimization were chosen as (1) energy tolerance of 5 × 10^{-5} eV, (2) maximum force tolerance of 0.1 eV $Å^{-1}$ and (3) Monkhorst−Pack k−point sampling of 2 × 2 × 2. The electron wave functions were expanded using plane waves with a cutoff energy of 400 eV. The NiO (200) slab was modeled by a 2 × 2 supercell, and a vacuum region of 15 Å was used to separate adjacent slabs. The Gibbs free energy (Δ*G*, 298 K) of reaction steps is calculated by:

$$\Delta G = \Delta E + \Delta ZPE - T\Delta S \quad \text{(S1)}$$

where Δ*E* is the adsorption energy, Δ*ZPE* is the zero-point energy difference and *T*Δ*S* is the entropy difference between the gas phase and adsorbed state. The entropies of free gases were acquired from the NIST database.

Molecular dynamics (MD) simulations were carried out using a force field type of universal. the electrolyte system was set up by randomly placing 1 000 H_2O, 50 NO_2 and 50 H molecules in the simulation box, with a catalyst model fixed perpendicular to the z−axis at the center of the simulation system. The electrolyte system was geometrically optimized by setting the convergence tolerance of 2.0 × 10^{-5} kcal/mol for energy and 0.001 kcal/mol/Å for force. The non−bond interaction was processed by Ewald method with accuracy of 1.0 × 10^{-5} Kcal/mol. After geometry optimization, the MD simulations were performed in an NVT ensemble (298 K) with the total simulation time of 1 ns at a time step of 0.2 fs. The radial distribution function (RDF) is calculated as

$$\mathrm{g}(r) = \frac{dN}{4\pi\rho^2 dr} \quad \text{(S2)}$$

where *dN* is the amount of $*NO_2$/*H in the shell between the central particle *r* and *r*+*dr*, *ρ* is the number density of $*NO_2$/*H.

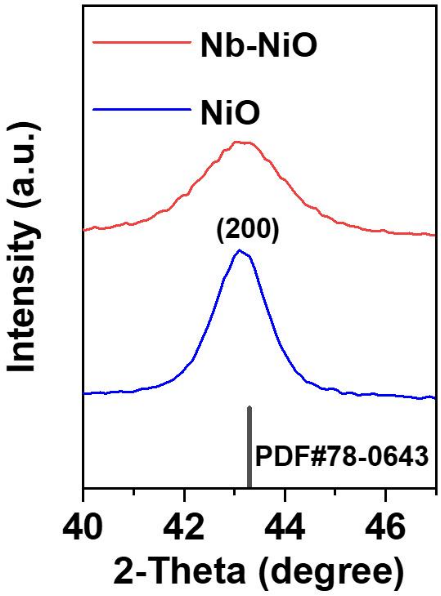

Fig. S1. Enlarged view for the XRD patterns of NiO and Nb−NiO.

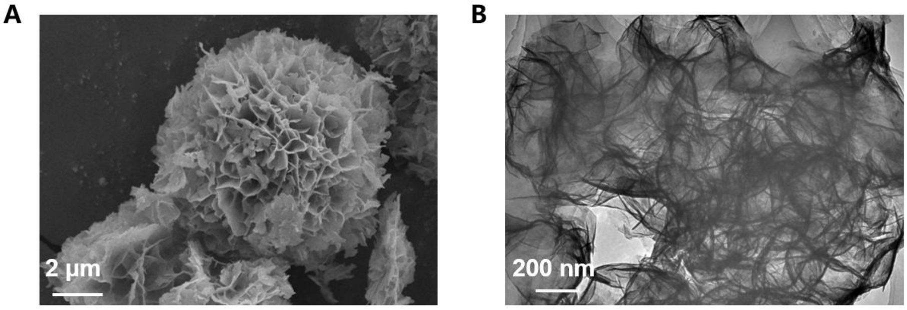

Fig. S2. Morphology of pristine NiO: (A) TEM image. (B) HRTEM image.

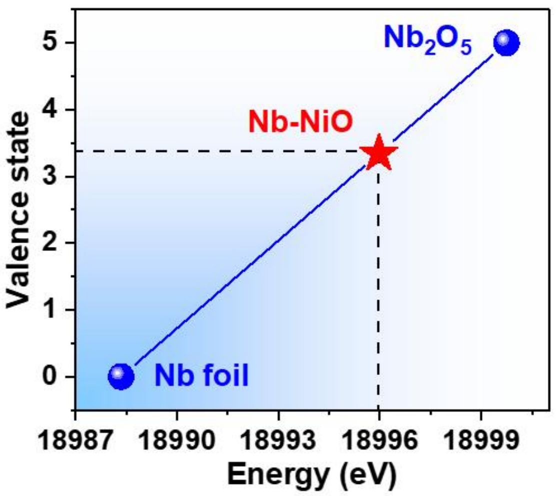

Fig. S3. XANES fitted average Nb valence state of Nb−NiO.

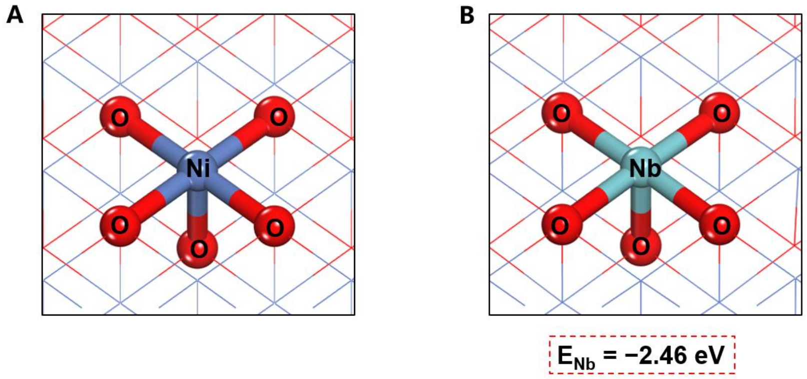

Fig S4. (A) Pristine NiO structure and (B) the formation energy of Nb−NiO.

Discussion:

The formation energy of Nb−NiO is calculated as[1]:

$$E = E(\text{Nb−NiO}) - E(\text{NiO}) - \mu_{Nb} + \mu_{Ni}$$

where *E* is the total energies of corresponding structures, μ is the chemical potential of corresponding atoms.

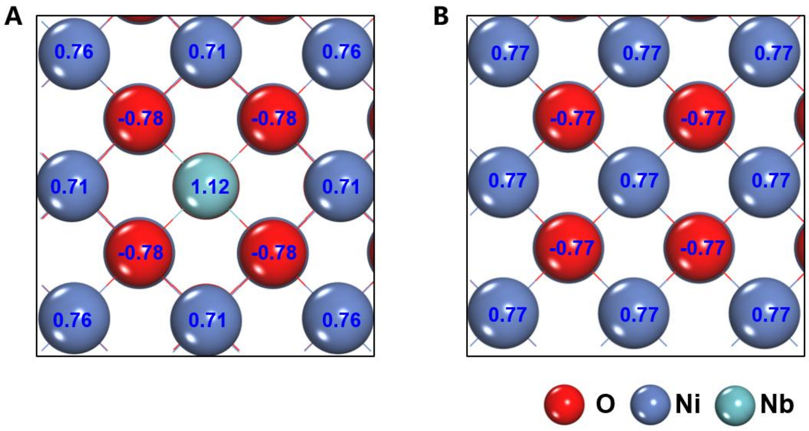

Fig. S5. Detailed charge analysis of (A) Nb−NiO and (B) NiO.

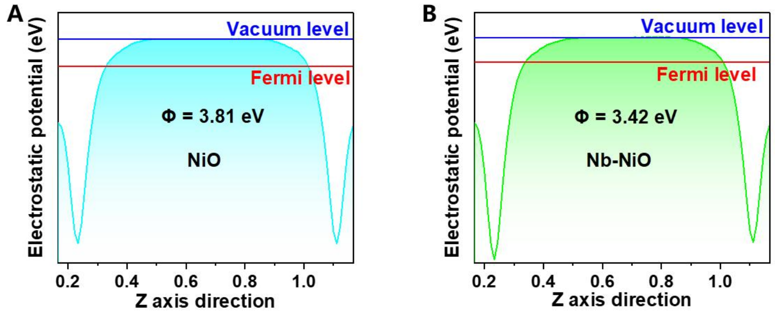

Fig. S6. Average potential profiles along c−axis direction for calculating the work functions of (A) NiO and (B) Nb−NiO.

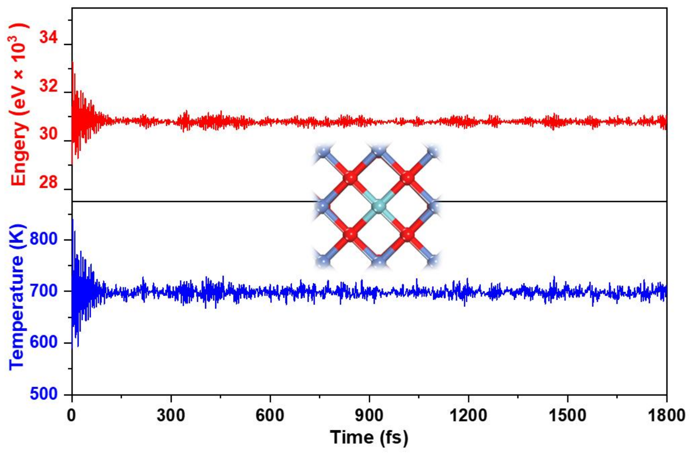

Fig. S7. Variations of energy and temperature at different AIMD simulation times (Inset is the geometric structure of Nb−NiO after 1800 fs of AIMD simulation at 700 K).

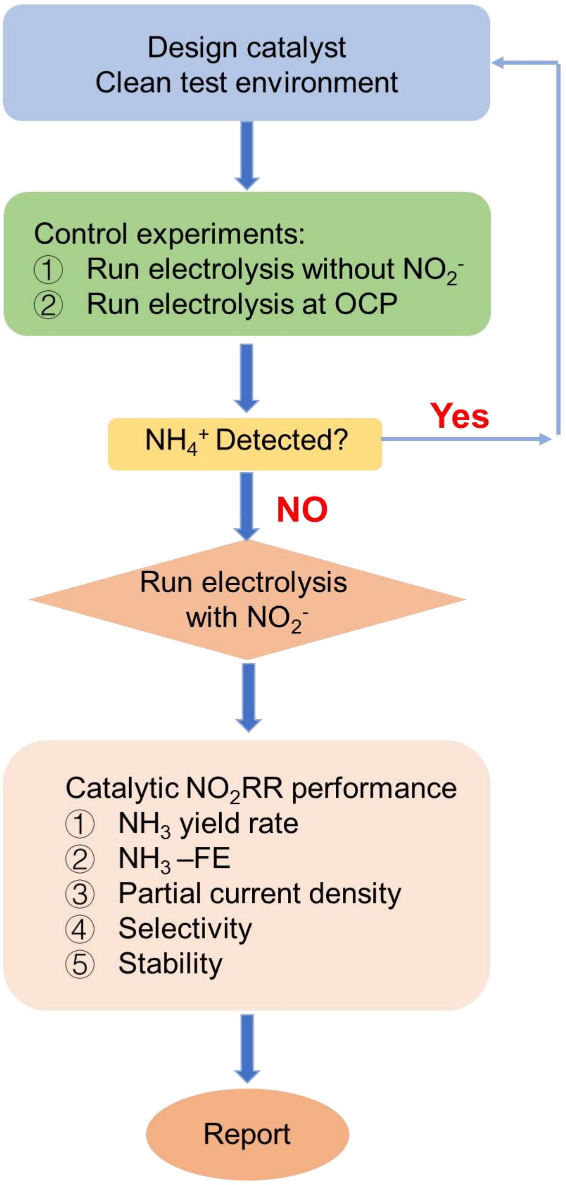

Fig. S8. Flow chart of the electrocatalytic NO_2RR measurement procedure.

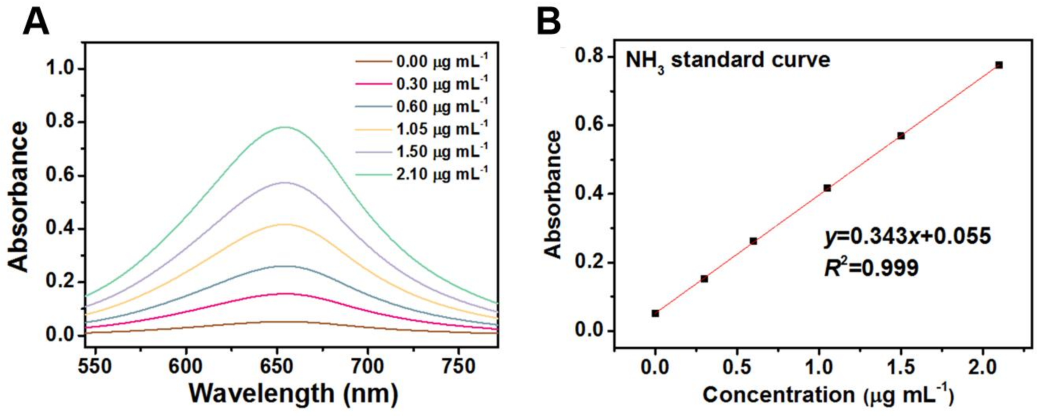

Fig S9. (A) UV-vis absorption spectra of NH_4^+ assays after incubated for 2 h at ambient conditions. (B) Calibration curve used for the calculation of NH_3 concentrations.

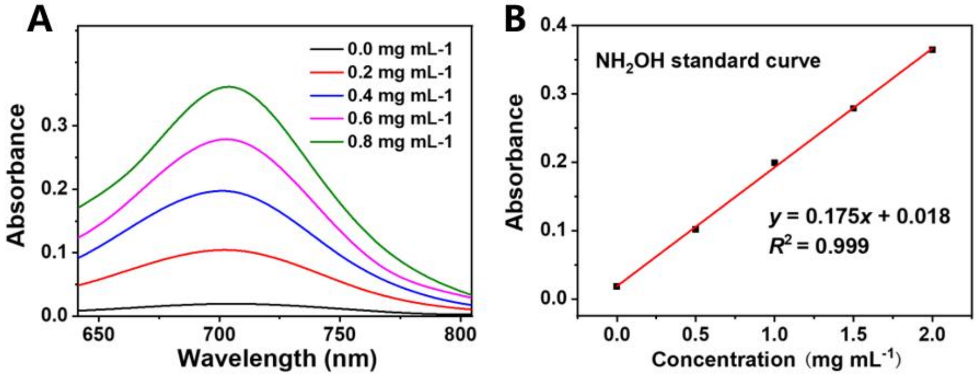

Fig. S10. (A) UV-vis absorption spectra of cooled NH_2OH assays. (B) Calibration curve used for the calculation of NH_2OH concentrations.

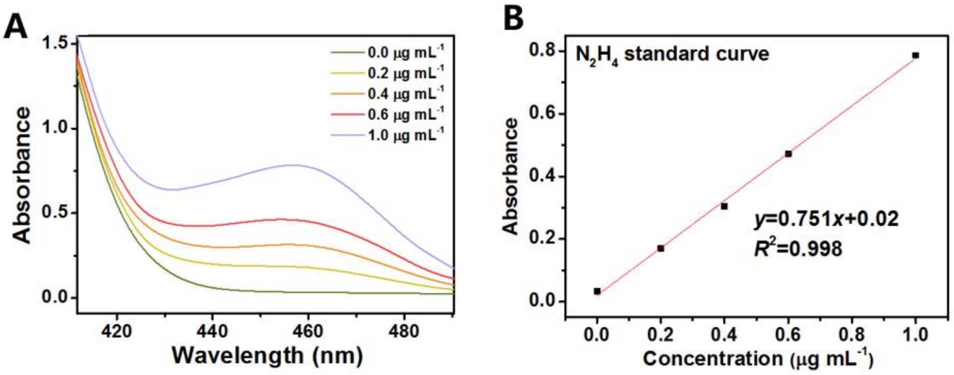

Fig. S11. (A) UV-vis absorption spectra of N_2H_4 assays after incubated for 20 min at ambient conditions. (B) Calibration curve used for calculation of N_2H_4 concentrations.

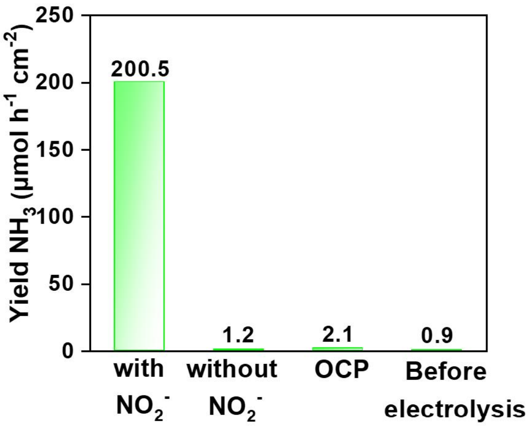

Fig. S12. Amounts of produced NH_3 on Nb−NiO under different conditions: (1) electrolysis in NO_2^-−containing solution at −0.6 V, (2) electrolysis in NO_2^-−free solution at −0.6 V, (3) electrolysis in NO_2^-−containing solution at open-circuit potential (OCP), (4) before electrolysis.

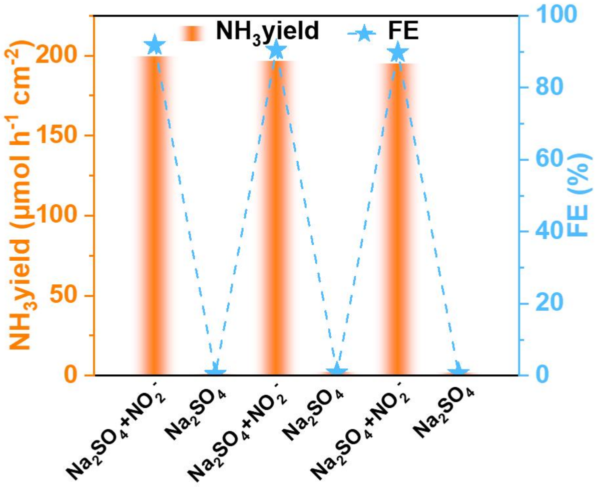

Fig. S13. Alternating experiments with and without NO_2^-.

Discussion:

The alternating cycling tests (Fig. S13) show that the NH_3 production is prominent during the NO_2^-−containing cycles, while the NO_2^-−free cycles show negligible NH_3 generation, thus confirming that the produced NH_3 is derived from the NO_2RR electrolysis on Nb−NiO.

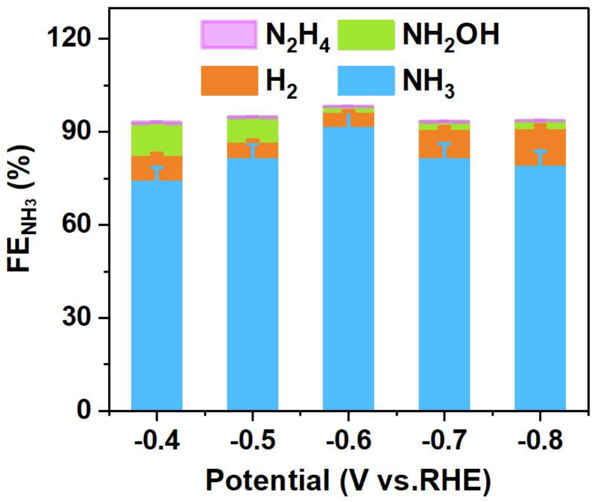

Fig S14. FEs of different products at various potentials after 0.5 h electrolysis.

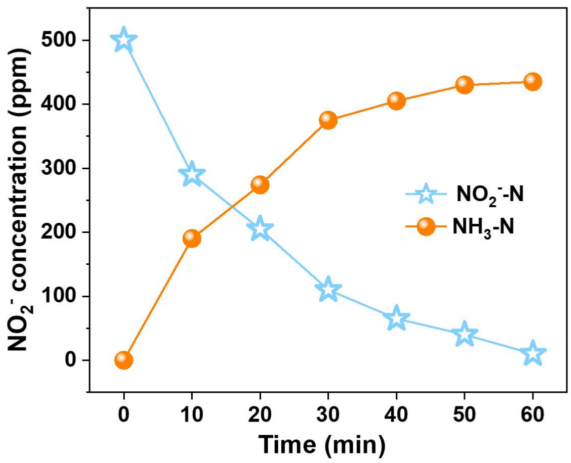

Fig. R15. The variations of NO_2^-−N and NH_3−N concentrations with NO_2RR electrolysis time on Nb−NiO at −0.6 V.

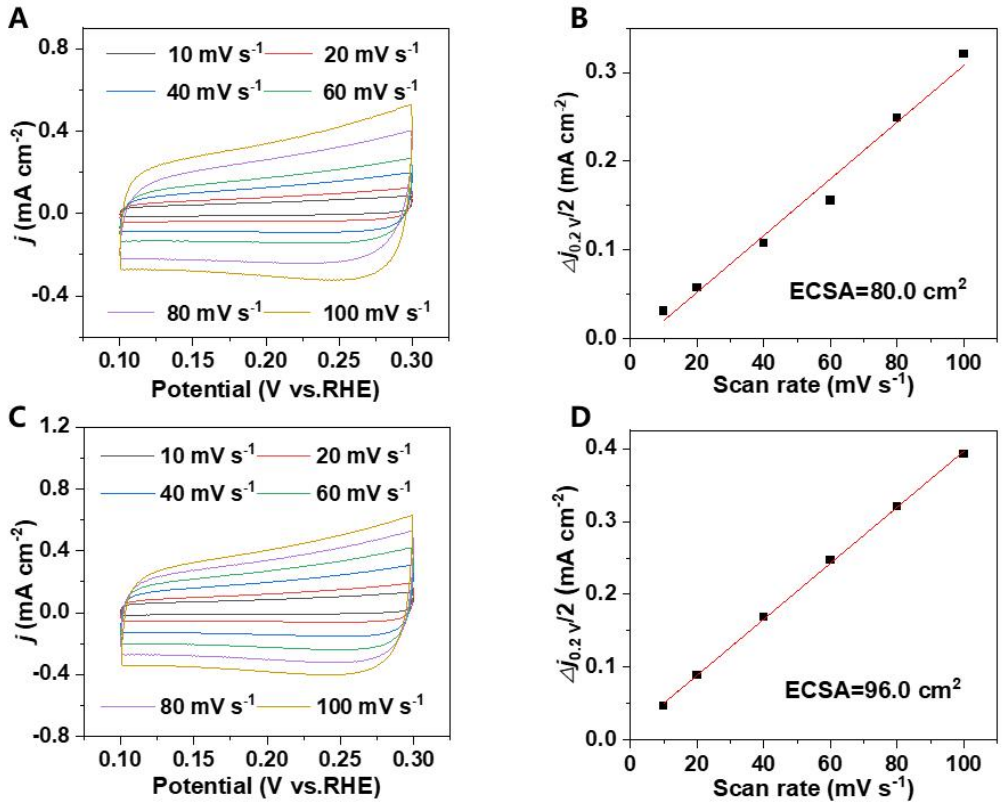

Fig. S16. CV measurements at different scanning rates and calculated ECSA for (A, B) NiO and (C, D) Nb−NiO.

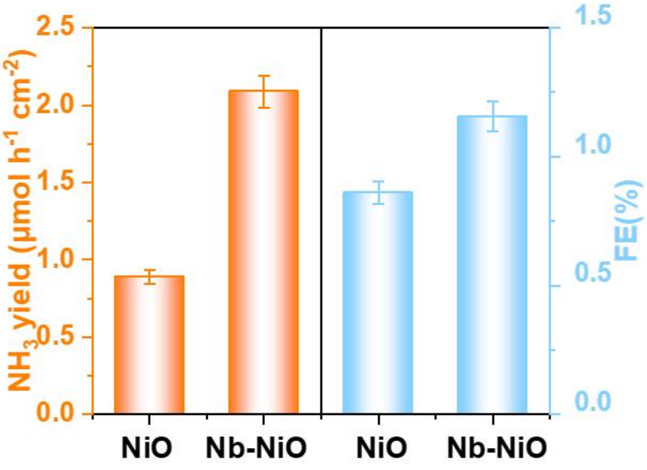

Fig. S17. Comparison of the ECSA-normalized NH_3 yield rates and FE_{NH3} between Nb−NiO and NiO at −0.6 V.

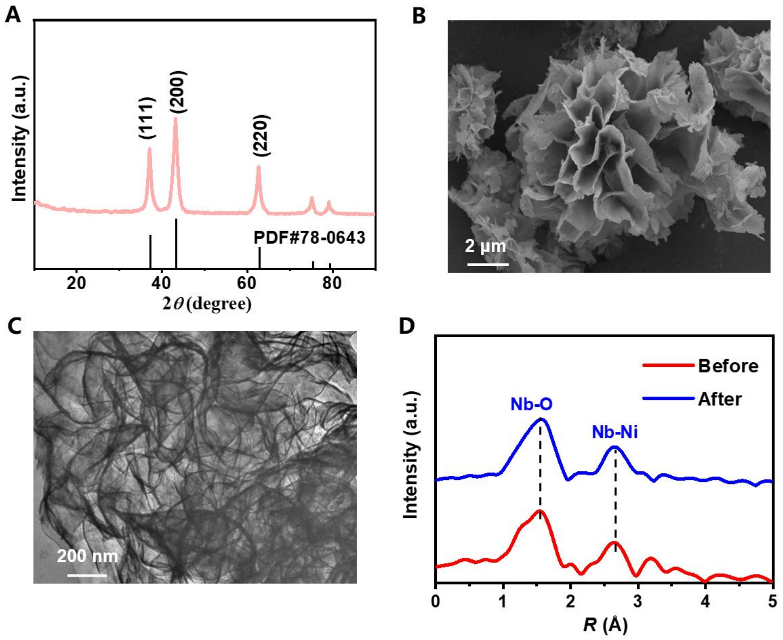

Fig. S18. Characterizations of Nb−NiO after electrolysis：(A) XRD pattern, (B) SEM image and (C) TEM image. (D) Nb K−edge EXAFS spectra of Nb−NiO before and after stability tests.

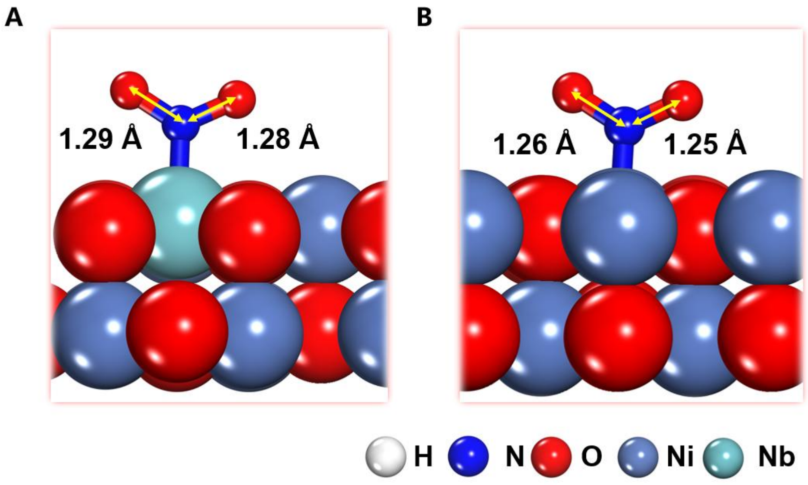

Fig. S19. The optimized structures of NO_2^- adsorption on (A) Nb−NiO and (B) NiO.

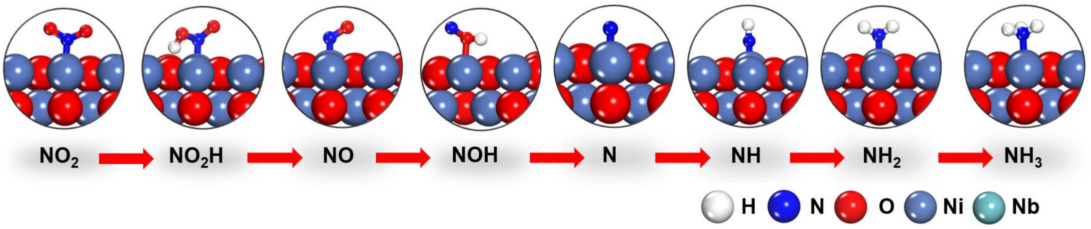

Fig. S20. The optimized atomic structures of the reaction intermediates on NiO.

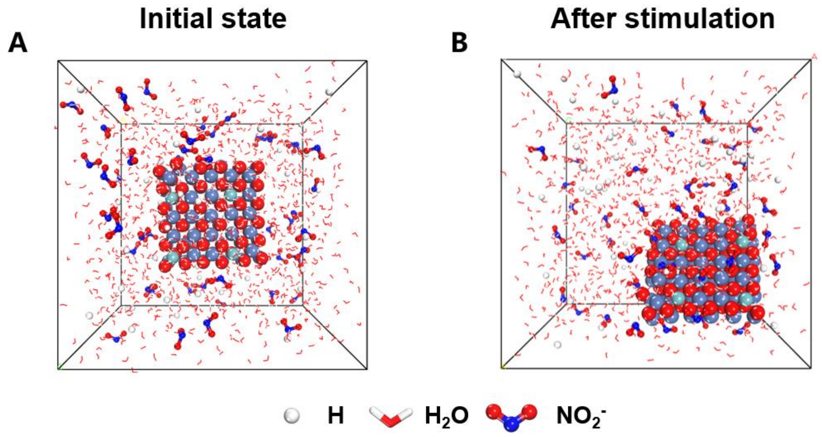

Fig. S21. (A) Initial and (B) simulated states of the dynamic process of NO_2^- and H adsorption on Nb−NiO.

Table S1 Nb K−edge EXAFS fitting results of Nb−NiO.

Sample	Shell	CN	R (Å)	σ^2 (10^{-3}Å)	ΔE_0 (eV)	R factor
Nb/NiO	Nb-Ni	8.2	3.15	7.2	-6.1	0.015
	Nb-O	5.3	2.04	4.8	1.9	
	Nb-O	0.6	1.91	2.6	-3.5	

CN is the coordination number, R is interatomic distance, σ^2 is Debye-Waller factor, ΔE_0 is edge-energy shift, R factor is used to value the goodness of the fitting.

Table 2 Comparison of the optimum NH_3 yield rate and FE_{NH3} for the recently reported state−of−the−art NO_2RR electrocatalysts at ambient conditions.

Catalyst	Electrolyte	NH_3 yield rate ($\mu mol\ h^{-1}\ cm^{-2}$ @ V vs. RHE)	FE_{NH3} (@ V vs. RHE)	Ref.
TiO_{2-x}/TP	(0.1 M NO_2^-)	464.6@ −0.7	92.7% @ −0.7	2
V-TiO_2/TP	0.1 M NaOH (0.1 M NO_2^-)	540.8@ −0.7	93.2%@ −0.6	3
Ni−NSA−V_{Ni}	0.1 M NaOH (200 ppm NO_2^-)	135.5@ −0.54	88.9%@ −0.54	4
Ni_2P/NF	0.1 M NaOH (200 ppm NO_2^-)	158.1@ −0.3	90.2%@ −0.3	5
Cu_3P Nanowire array	0.1 M PBS (0.1 M NO_2^-)	95.7@ −0.5	91.2%@ −0.5	6
CoP nanoarray	0.1 M PBS (500 ppm NO_2^-)	133.0@ −0.2	90.2%@ −0.2	7
$[Co(DIM)Br_2]^+$	0.1 M $NaNO_2$	/	88.0%	8
$Cu_{80}Ni_{20}$	0.1 M NaOH (20 mM NO_2^-)	/	87.6%@ −0.335	9
Co tripeptide complex	0.1 M MOPS (0.1 M NO_2^-)	1.1@ −0.29	90%@ −0.29	10
MnO_2 nanoarrays	0.1 M Na_2SO_4 (0.2 M NO_2^-)	0.11@ −0.885	6%@ −0.885	11
**Nb/NiO**	**0.5 M Na_2SO_4 (0.1 M NO_2^-)**	**200.6@ −0.6**	**92.4%@ −0.6**	**This Work**

References

1. Chu, K., Wang, J., Liu, Y.-p., Li, Q.-q., and Guo, Y.-l. (2020). Mo-doped SnS2 with enriched S-vacancies for highly efficient electrocatalytic N2 reduction: the critical role of the Mo–Sn–Sn trimer. J. Mater. Chem. A *8*, 7117-7124. 10.1039/D0TA01688H.
2. Zhao, D., Liang, J., Li, J., Zhang, L., Dong, K., Yue, L., Luo, Y., Ren, Y., Liu, Q., Hamdy, M.S., et al. (2022). A TiO_{2-x} nanobelt array with oxygen vacancies: an efficient electrocatalyst toward nitrite conversion to ammonia. Chem. Commun. *58*, 3669-3672.
3. Wang, H., Zhang, F., Jin, M., Zhao, D., Fan, X., Li, Z., Luo, Y., Zheng, D., Li, T., Wang, Y., et al. (2023). V-doped TiO_2 nanobelt array for high-efficiency electrocatalytic nitrite reduction to ammonia. Mater. Today Phys. *30*, 100944.
4. Wang, C., Zhou, W., Sun, Z., Wang, Y., Zhang, B., and Yu, Y. (2021). Integrated selective nitrite reduction to ammonia with tetrahydroisoquinoline semi-dehydrogenation over a vacancy-rich Ni bifunctional electrode. J. Mater. Chem. A *9*, 239-243.
5. Wen, G., Liang, J., Zhang, L., Li, T., Liu, Q., An, X., Shi, X., Liu, Y., Gao, S., Asiri, A.M., et al. (2022). Ni_2P nanosheet array for high-efficiency electrohydrogenation of nitrite to ammonia at ambient conditions. J. Colloid Interface Sci. *606*, 1055-1063.
6. Liang, J., Deng, B., Liu, Q., Wen, G., Liu, Q., Li, T., Luo, Y., Alshehri, A.A., Alzahrani, K.A., Ma, D., and Sun, X. (2021). High-efficiency electrochemical nitrite reduction to ammonium using a Cu_3P nanowire array under ambient conditions. Green Chem. *23*, 5487-5493.
7. Wen, G., Liang, J., Liu, Q., Li, T., An, X., Zhang, F., Alshehri, A.A., Alzahrani, K.A., Luo, Y., Kong, Q., and Sun, X. (2022). Ambient ammonia production via electrocatalytic nitrite reduction catalyzed by a CoP nanoarray. Nano Res. *15*, 972-977.
8. Xu, S., Kwon, H.-Y., Ashley, D.C., Chen, C.-H., Jakubikova, E., and Smith, J.M. (2019). Intramolecular hydrogen bonding facilitates electrocatalytic reduction of Nitrite in aqueous solutions. Inorg. Chem. *58*, 9443-9451.
9. Mattarozzi, L., Cattarin, S., Comisso, N., Guerriero, P., Musiani, M., Vázquez-Gómez, L., and Verlato, E. (2013). Electrochemical reduction of nitrate and nitrite in alkaline media at CuNi alloy electrodes. Electrochim. Acta *89*, 488-496.
10. Guo, Y., Stroka, J.R., Kandemir, B., Dickerson, C.E., and Bren, K.L. (2018). Cobalt metallopeptide electrocatalyst for the selective reduction of Nitrite to Ammonium. J. Am. Chem. Soc. *140*, 16888-16892.
11. Wang, R., Wang, Z., Xiang, X., Zhang, R., Shi, X., and Sun, X. (2018). MnO_2 nanoarrays: an efficient catalyst electrode for nitrite electroreduction toward sensing and NH_3 synthesis applications. Chem. Commun. *54*, 10340-10342.
